# The gulf of cross-disciplinary research collaborations on global river basins is not narrowed

**DOI:** 10.1007/s13280-022-01716-0

**Published:** 2022-03-23

**Authors:** Yongping Wei, Shuanglei Wu

**Affiliations:** 1 School of Earth and Environmental Sciences, The University of Queensland, Room 517, Chamberlain Building, Brisbane, 4072 Australia; 2 School of Earth and Environmental Sciences, The University of Queensland, Room 427, Chamberlain Building, Brisbane, 4072 Australia

**Keywords:** Complex knowledge system, Cross-disciplinary research collaboration, Earth system science, Global river basin governance

## Abstract

Using publications in the Web of Science database (WoS), this study investigates the research collaboration on the top 95 most researched global river basins since 1900. The links of both the disciplines involved and the management issues studied between the biophysical, economic, societal, climatic and governance sub-systems of these river basins were examined. We found that research collaborations were dominated within the biophysical sub-system (65.3%) since the knowledge predevelopment period (1900–1983), with continuous increases (by 18.5%) during the rapid development (1984–2000) and the stabilisation (12.9% increase) (2001–2017). However, research collaborations related to the societal sub-system remained marginalised (varied at about 1%), while those related to the governance sub-system expanded in issues studied (32.8%) but were not supported by the core governance disciplines (3.4%). The key findings explained why global river basins are degraded from the perspective of knowledge development and they can assist the strategic planning and management of scientific research for improving governance capacity in modifying the relationship between human and nature on river basins in the Anthropocene. Tackling challenges in the Anthropocene requires transformation of the current pattern of knowledge development, a revolution in the governance of science.

**Supplementary Information:**

The online version contains supplementary material available at 10.1007/s13280-022-01716-0.

## Introduction

Humans have extensively intervened in the Earth system cycles, resulting in the transition of our planet into a human dominated era—the Anthropocene (Crutzen and Stoermer 2000). To tackle complex and ill-defined problems in the Anthropocene, cross-disciplinary research collaborations have long been recognised (Berkes et al. 2008; Ostrom 2009; McCurley and Jawitz 2017; Kinnebrew et al. 2020). Since 1980s, global research initiatives such as the establishment of NASA’s Earth Science Advisory Committee (ESAC), the Earth System Science Partnership (ESSP), the International Human Dimensions Programme on Global Environmental Change (IHDP), and the Earth System Governance Project (a core project to the Future Earth global research network) have endeavoured to promote cross-disciplinary research collaborations. However, there is yet to be extensive, systemic investigation on such research collaborations in river basin studies, compromising our capacity to strategically planning scientific research on this globally significant field.

Increasingly intertwined interactions between human and nature have shifted the way cross-disciplinary research is conducted. The linear model of scientific research within academic institutes is being replaced by non-linear knowledge co-production models. The “Triple Helix”, by enhancing dynamic collaboration between university, industry and government has been advocated as a typical non-linear knowledge production model (Etzkowitz and Leydesdorff 1998). Recently, the “Quadruple Helix” is proposed to frame the “Triple Helix” in a culture-based social environment, and the “Quintuple Helix” further embeds the “Quadruple Helix” into the natural environment (Carayannis and Campbell 2009; Galvao et al. 2019). These new knowledge models require the continuous involvement of the whole disciplinary spectrum, ranging from natural sciences to social sciences and humanities (Holm et al. 2013). Underlying this is that science is increasingly recognised as a complex and dynamic network within which different disciplines “knit, weave and knot together into an overarching scientific fabric” (Latour 1987; Shi et al. 2015) to provide system solutions for increasingly intertwined problems in the Anthropocene.

River basins are basic components of the Earth system. They represent the logical management units of water cycles which link to other cycles (e.g., nutrients, energy, carbon) of the Earth system. Thus, all human decisions and governance actions on river basins have interdependent biophysical, economic, societal, and climatic implications (Newson 2008). Two thirds of global river basins are degraded and are expected to face further environmental deterioration in the Anthropocene (McDonald et al. 2016; Grill et al. 2019). In the past decades, 217 disciplines of the total 254 disciplines in the Web of Science database have been involved in river basin studies (Wu et al. 2021), reflecting the continued advances in cross-disciplinary research on coupled human-natural systems (Ferraro et al. 2019), socio-hydrological systems (Pan et al. 2018; Gain et al. 2020), and natural-based solutions (Thorslund et al. 2017; Pan et al. 2021). Thus, the river basin is a typical case to investigate research collaboration on the Earth system.

This study aims to reveal blind spots and weakness of research collaboration on global river basins by examining the interconnectedness of both the disciplines involved and the management issues studied between biophysical, economic, societal, climatic and governance sub-systems of a river basin. The scientific publications indexed in the Science Citation Index (SCI) and the Social Sciences Citation Index (SSCI) of the Web of Science database (WoS) since 1900 were used as the data source. The key findings from this study are expected to explain why global river basins are degraded from the perspective of knowledge development and to assist the strategic planning and management of scientific research for improving governance capacity in modifying the relationship between human and nature on river basins under climate change in the Anthropocene.

## Materials and methods

### Defining a river basin system as five interactive sub-systems

**Fig. 1:**
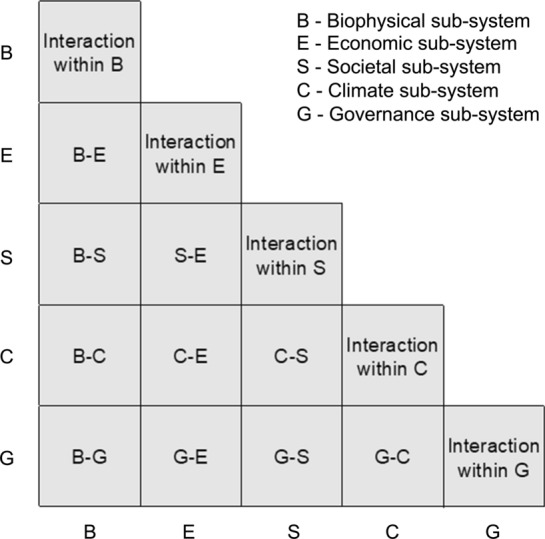
Defining the interactive sub-systems of a river basin system

We adopted a system-based approach by recognising the diverse interactions and bi-directional feedback between system components and sub-systems (Malerba 2007; Wei et al. 2018). We firstly defined a river basin as a complex system consisting of biophysical and societal sub-systems to capture both natural and societal processes and feedback between them, thus to reflect the human-nature relationship in the Anthropocene. The biophysical sub-system includes all the physical, biological, and chemical processes and their co-evolutionary dynamics. As the climatic sub-system is the cornerstone of the Earth System and is considered as the external driver of all other sub-systems due to its substantial impacts on the global environment, we separated it from the biophysical sub-system. Furthermore, we considered the societal sub-system as three parts: the economic sub-system which covers the economic/material processes (commercial, industrial, and technological/engineering development) that have interactive influences on the biophysical sub-system; the societal sub-system which contains all the societal/immaterial processes (culture, religion, value judgments, political concerns) that drive human behaviours (DeCaro et al. 2017); and the governance sub-system which is defined as the institutional arrangements and management action. It, on one hand, has to coordinate the interactive economic and biophysical sub-systems (Pahl-Wostl et al. 2010); on the other hand, is constructed and deeply rooted in the societal sub-system (Smith and Stirling 2010). So, we defined a river basin system as five sub-systems: the biophysical (B), economic (E), societal (S), governance (G), and climatic (C) sub-systems (Fig. 1). The purpose of such a definition is not to question the existing classification system, but to help examine the research collaboration for modifying the relationship between human and nature under climate change in the Anthropocene, which is the aim of this study. It should be noted that those cross-disciplinary, multi-disciplinary and inter-disciplinary studies (e.g. eco-hydrology, socio-hydrology, hydro-economics) were considered as direct research collaborations among the sub-systems in this definition. Using this definition, we can reveal blind spots and weakness of research collaboration within and between any sub-systems for assisting the strategic transformation of scientific research on river basins in the Anthropocene.

### Data source

**Fig. 2:**
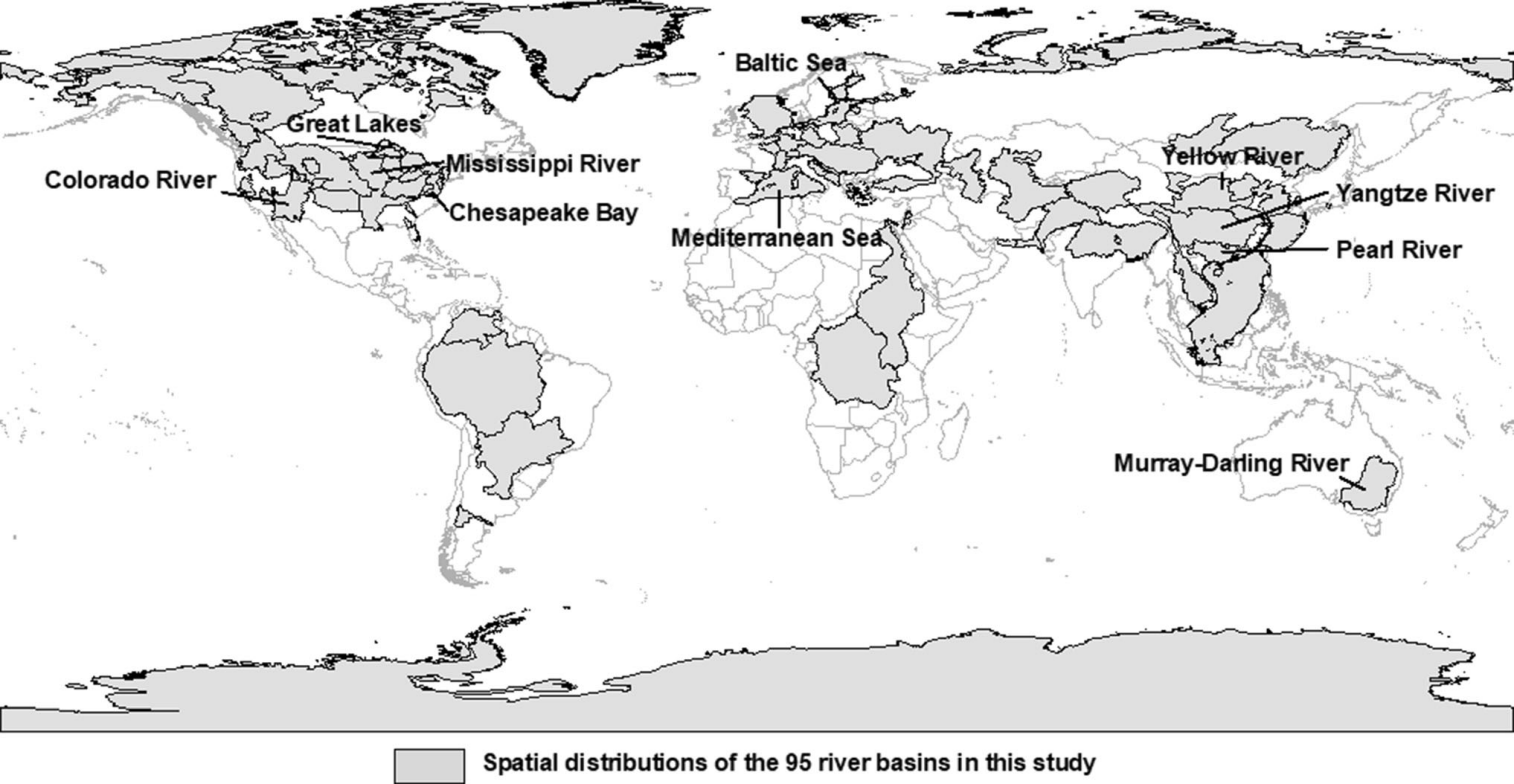
The spatial location of the most researched 95 river basins, labelling the top 10 most published river basins

Scientific publications indexed in the Science Citation Index (SCI) and the Social Sciences Citation Index (SSCI) of the Web of Science database (WoS) were used as the data source in this study. As one of the largest scientific publication databases, WoS archives over 12 000 top-tier international and regional journals, covering all areas of natural, social and humanity sciences (Rousseau et al. 2019). As our study focuses on research collaboration related to river basins, the Boolean search equation: “catchment” OR “drainage basin” OR “drainage area” OR “hydrographic basin” OR “hydrological basin” OR “river basin” OR “valley” OR “watershed” OR “wetland” was used to identify the relevant publications in the database to ensure the articles retrieved have the most comprehensive coverage on river basin studies. The document type was specified to be “Article” and in “English” language. The “Title”, “Abstract” and “Keywords” sections of each publication in the WoS database were screened to ensure sufficient coverage of each publication’s contents. Publications related to smaller spatial units (e.g., sub-catchment, delta, wetland) were manually screened and added to the river basins they were affiliated to. Duplicate and irrelevant publications were removed. The top 100 river basins with the largest number of publications were collected, covering main river basin studies in the world. A total of 95 river basins were finally used in analysis after river basins with data issues were removed (Fig. 2). The study period was from 1900, when the earliest publication was archived in the WoS, to 2017.

### Measuring the research collaboration within and between sub-systems of a river basin system

Discipline knowledge is typically recognised as a system. It represents ordered knowledge of phenomena and the rational study of the relations between the concepts in which those phenomena are expressed (Dampier 1944). In our context, we defined knowledge development regarding a river basin as a co-evolutionary process involving scientific disciplines and management issues, each of which have their respective evolutionary dynamics. We investigated the research collaboration from the perspective of both the disciplines and management issues and assessed if they are properly matched in terms of their respective sizes and changes over time.

Academic journals indexed in the WoS are already classified according to the ISI Subject Category,^1^ which includes 254 disciplines under five major Research Areas: arts and humanities, life sciences and biomedicine, physical sciences, social sciences, and technology.^2^ Thus, publications retrieved on river basins have been unambiguously classified into the disciplines of the journals. As the disciplines specifically labelled as cross-disciplinary, multi-disciplinary and inter-disciplinary only accounted for 1% of all collaboration, we did not separate them from other collaborations. We mapped all disciplines into the five sub-systems of a river basin system (Fig. 1) according to the following principles (refer to Supplementary Information Table A1 for the disciplines grouped under each of the five sub-systems):

- All disciplines in physical sciences except those related to climate were grouped to the biophysical sub-system;
- All disciplines related to economic activities, technological and engineering development were grouped to the economic sub-system;
- All disciplines from social sciences, arts and humanities related to understanding of human behaviours and the society as a whole (except those on governance) were included in the societal sub-system;
- Meteorology and Atmospheric Science and multidisciplinary science related to climate were included in the climatic sub-system;
- All disciplines related to regulation, policy, management, and governance were included in the governance sub-system; and
- Each discipline was assigned to one sub-system exclusively.

Since key words can directly express the research topic (management issues in the context of this study) and their co-occurrence relations provide insights into the structure and development of knowledge (Cheng et al. 2020), we extracted key words from the titles, abstracts, and author-selected keywords sections in these publications to ensure the most comprehensive representation of the research topics. Pre-processing of these keywords were conducted using the Natural Language Processing (NLP) module in the Derwent Data Analyzer,^3^ which removed duplicates, special characters and meaningless stop words and tokenised remaining ones into meaningful key words. These key words were then grouped into the five sub-systems of a river basin according to the principles below (refer to Supplementary Information Table A2 for the key words grouped under each of the five sub-systems):

- Key words related to changes of the physical, chemical, ecological, biological processes and their impacts were grouped into the biophysical sub-system;
- Key words related to the economic activities (e.g., engineering, technology, material, energy, urban and rural development, health, and recreations) were included into the economic sub-system;
- Key words related to functional elements (e.g., education, population), power relations (e.g., equality, power, crime) and symbolic attachment (e.g., religion, media and communication, citizenship, value in society) of a society were included in the societal sub-system;
- Key words related to the changes in climate including temperature, precipitation, greenhouse gasses, other climatic extremes were included as the climatic sub-system;
- Key words related to governance activities including institutions (e.g., organisations and agencies), policy instruments (e.g., tax and subsidy, permit and certification, trading, and entitlement), measures (e.g., monitoring, mapping, and tool), and management in general (e.g., governance, management and control, plan, and strategy) were grouped into the governance sub-system; and
- Each key word was assigned to only one sub-topic and sub-system, respectively.

Research collaborations within and between the five sub-systems were measured using network analysis. Connections for the respective discipline and key word systems were established based on the co-word principle (i.e., if they are linked to the same discipline/key word in a publication) (Callon et al. 1983). We used *collaboration* (*C*) (defined as the ratio of actual connections to the potential maximum connections in the network) to measure the degree of research collaboration establishment. C between one sub-system i and another sub-system j (for both discipline and key word networks) can be calculated using Eq. 1:

1 $${C}_{ij}=\frac{{\text{no.}}\;{\text{of}}\;{\text{existing}}\;{\text{connections}}\;{\text{between}}\, i,j}{{\text{Maximum}}\;{\text{potential}}\;{\text{connections}}}\times \;100\%$$

By taking into consideration the number of publications as weighting factors between sub-systems, we defined the *collaboration strength* (*CS*) between any sub-system i and j using the following equations:

2 $${CS}_{ij}=\frac{{\text{no.}}\;{\text{of}}\;{\text{existing}}\;{\text{connections}}\;{\text{between}}\, i,j}{{\text{Maximum}}\;{\text{potential}}\;{\text{connections}}}\times {\text{normalised}}\left({\text{sum}}\;{\text{of}}\;{\text{publications}}\;{\text{between}}\, i,\;j\right)\times \;100\%$$

The collaboration strength (CS) for each river basin is calculated using:

3 $${CS}_{n}=\frac{{\text{no.}}\;{\text{of}}\;{\text{existing}}\;{\text{connections}}}{{\text{Maximum}}\;{\text{potential}}\;{\text{connections}}}\times {\text{normalised}}\left({\text{sum}}\;{\text{of}}\;{\text{publications}}\;{\text{in}}\; n\right)\times\; 100\%$$

where n represents a particular river basin.

To assess the changes in C and CS with time, we divided the study period 1900–2017 into different temporal periods based on the total number of publications retrieved in time. Three temporal periods were defined: pre-development, rapid development, and stabilisation. The change point detection method was used to identify the divisions between different periods when there were abrupt changes in the growth rates of scientific publications in year. 

## Results

### Development of river basin publications in terms of disciplines and management issues

**Fig. 3:**
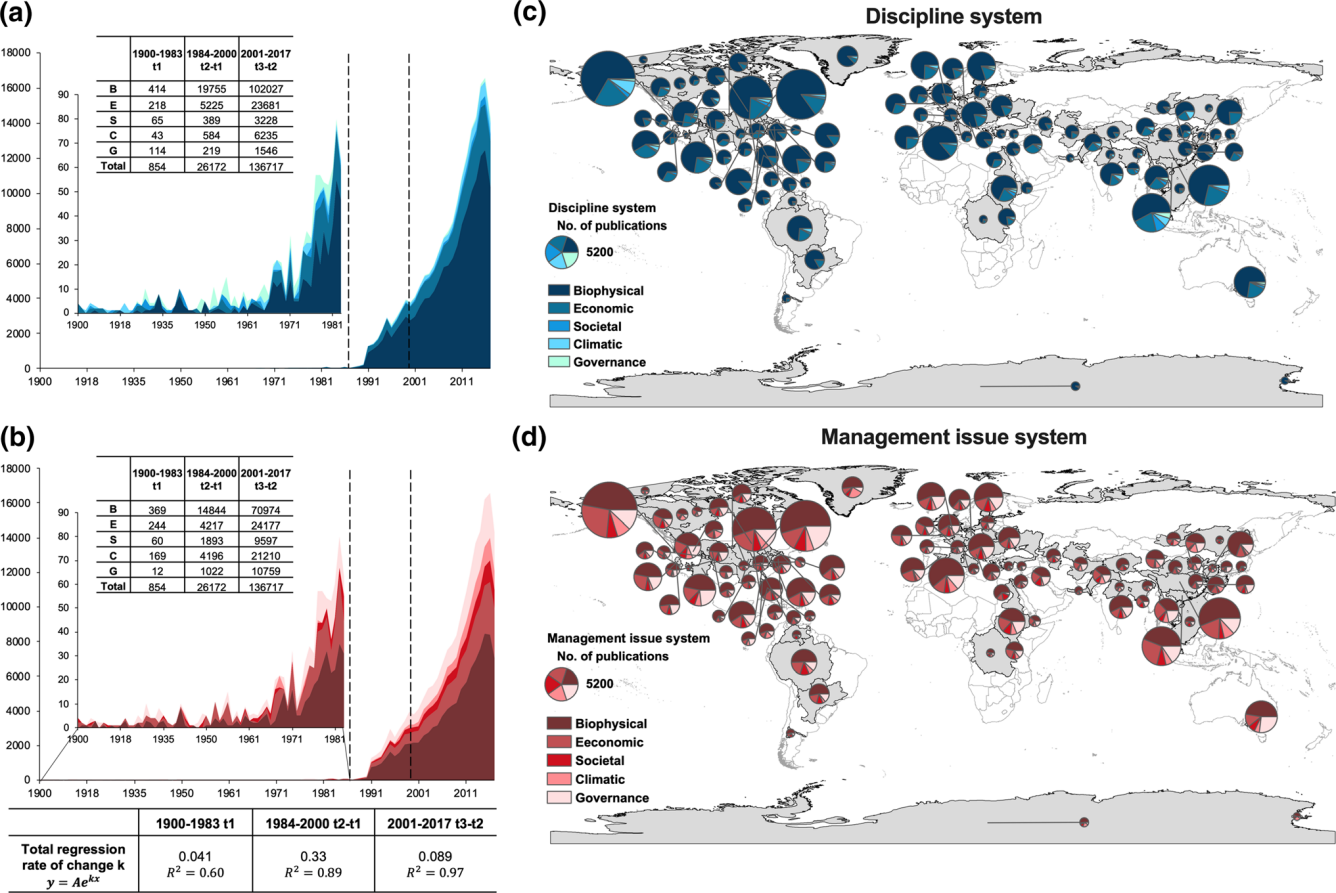
The temporal development of **a** the overall discipline system and **b** the overall management issue system; the spatial development of **c** the discipline systems in global river basins and **d** the management issue systems in global river basins

Three temporal periods of publications on river basin were identified: pre-development (1900–1983), rapid development (1984–2000), and stabilisation (2000–2017) (Fig. 3a, b). While all three periods were characterised by power-law relations in publications, each period were identified when there was significant changes in growth rates. The rate of growth during the pre-development period (1900–1983) was the lowest (0.04), with maximum publication per year lower than 100. During this period, the discipline system was most related to the biophysical sub-system (48%), followed by the economic sub-system (26%). The top five disciplines from the biophysical sub-system were Environmental Sciences, Water Resources, Multidisciplinary Geosciences, Geochemistry and Geophysics, and Marine and Freshwater Biology. Similar publication distributions were also observed in the management issues, with the biophysical sub-system having 43% and the economic sub-system having 29%. The top 5 management issues in the biophysical sub-system were pollution and treatment, water scarcity and availability, ecological degradation and restoration, salinity and alkalinity, and erosion and sedimentation.

Rapid expansions in the number of publications took place during 1984–2000, with over 8 times increase in the publication growth rate. This was most evident since the 1990s, when the number of publications per year exceeded 1000. The greatest increase in publication (19 755) was observed in the biophysical sub-system for the disciplines, which consisted of over 75% of total growth. For the top five disciplines in the biophysical sub-system, Ecology replaced Geochemistry and Geophysics. For the management issues, although the biophysical system was still the focus (increased by 14 844 publications and 57% of total growth), all other sub-systems also received over 1000 publications increases. New inclusion in the top 5 issues in the bio-physical sub-system were “flood and drought” and “other hazards (e.g., earthquakes)”, replacing “water scarcity” and “salinity and alkalinity”. Hydropower development became one of the top economic issues.

During the stable development period (2000–2017), the publications on river basins continued to grow and exceeded 10 000 publications per year since 2013. However, the rate of increase was much slower, being only 2 times greater than the pre-development period. Most of the increase in publications continued to be relevant to the biophysical sub-system (75% of total growth) and the economic sub-system (17% of total growth) in the discipline system, with less than 10% publications on the climatic, societal, and governance sub-systems. The top disciplines in the biophysical sub-system remained largely unchanged, whereas there were rapid increases in new disciplines in other sub-systems, including Industrial Engineering (E), History of Social Science and Communication (S) and Industrial relations and Labour (G), which contributed to a 40% growth in the number of disciplines developed in the overall discipline system. On the other hand, the focuses of management issues were even more diverse. While the proportional growths for the biophysical (52%) and economic (18%) sub-systems remained relatively consistent, the climatic sub-system also received relatively high level of publications (growth proportion ranged between 15 and 20% in time). The top five management issues in the biophysical sub-system remained largely unchanged, with water scarcity replacing other hazards. More new issues raised for the other sub-systems, with the top three being agriculture and irrigation, urban issues, and hydropower (E), population, value, and history (S), climate change, other climatic extreme, and sustainability (C), management and control, risk and impact assessment, and plan and strategy (G).

From a spatial perspective (Fig. 3c, d), the top three most published river basins were the Yangtze River, the Great Lakes, and the Mississippi River. Among the top 10 rivers (i.e., the Yangtze River, the Great Lakes, the Mississippi River, the Yellow River, the Pearl River, the Mediterranean Sea, the Colorado River, the Murray-Darling River, the Baltic Sea, and the Chesapeake Bay) which contributed to nearly 40% of total publications on river basins, 4 are in North America, 3 in Asia, 2 in Europe and 1 in Oceania. Rivers in Oceania, Africa and the two Poles (e.g., Lake Victoria, the Beaufort Sea) tended to receive fewer research interests. It was a global tendency for river basins to have the most focus on the biophysical, the economic and the climatic sub-systems, except the Murray-Darling River basin which had a stronger focus on the societal and the governance sub-systems relative to the climatic sub-system. On the other hand, although the management issues for all river basins were mostly related to the biophysical sub-system, American, Asian, and African river basins (over 80%) had a secondary focus on the economic sub-system, whereas for European and Oceania River basins (over 70%) the focus on the governance sub-system was put before the economic sub-system.

### Temporal change of research collaborations on disciplines

**Fig. 4:**
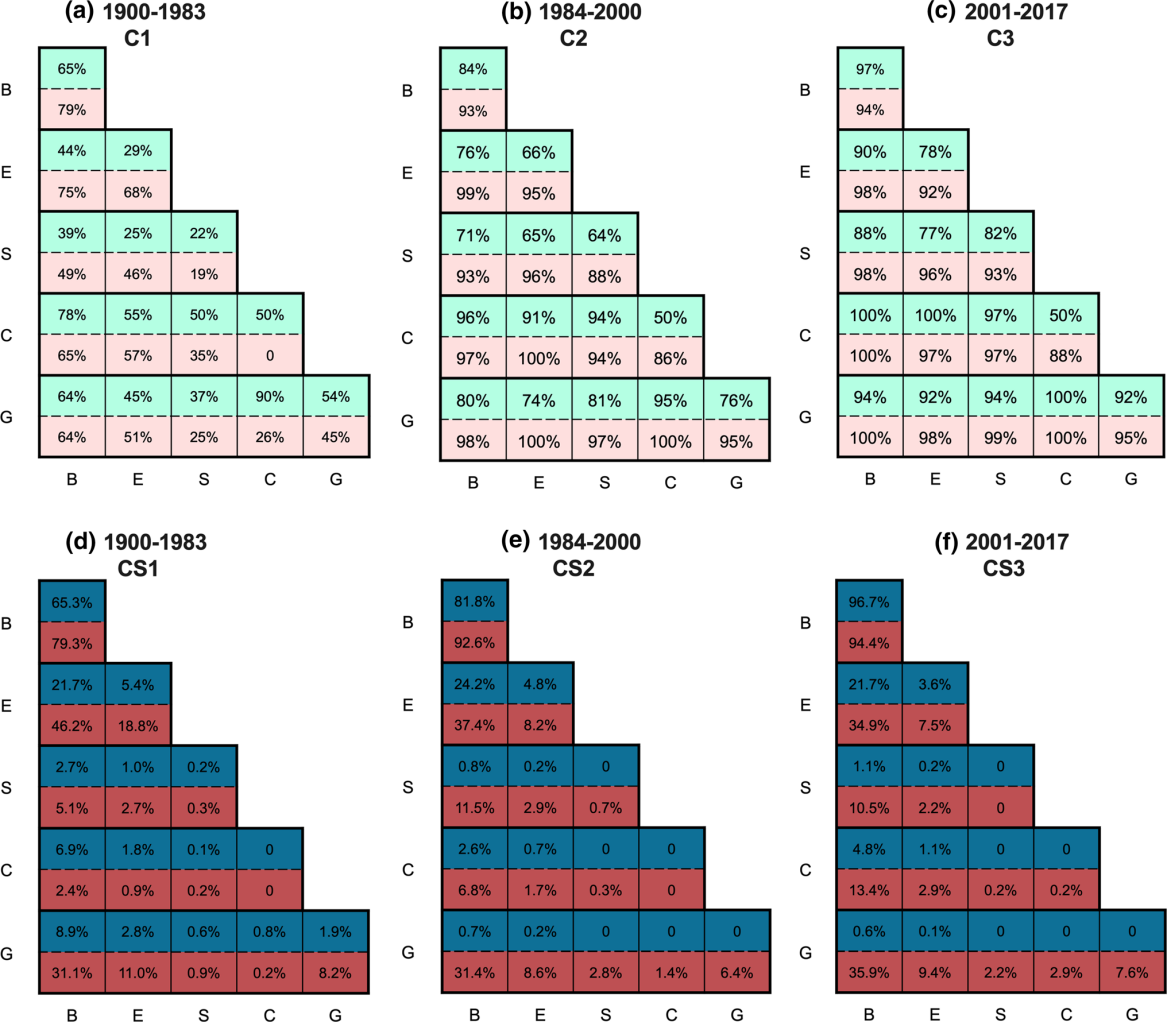
Comparisons between the disciplines (top half cells) and management issues (bottom half cells) for collaboration (C) during **a** 1900–1983, **b** 1984–2000, and **c** 2001–2017; comparisons for collaboration strengths (CS) during **d** 1900–1983, **e** 1984–2000, and **f** 2001–2017

For the discipline system (Fig. 4, and for Supplementary Information B for most connected disciplines in the sub-systems), research collaborations (C) within and between most of sub-systems had been established during the initial 1900–1983 period. The strongest were between the biophysical and climatic (*C* = 78%) and within the biophysical (*C* = 65%) sub-systems. Collaborations within the economic sus-system (*C* = 29%), within the societal sub-system (*C* = 22%), and between the societal and economic sub-systems (*C* = 25%) were the weakest. Taking the number of publications in each sub-system into account, the highest collaboration strength (CS) was within the biophysical sub-systems during this period (CS = 65.3%), most dominantly between Environmental Science and Water Resources disciplines. 75% of the total CSs within the biophysical sub-system was contributed by its top 10 disciplines. Due to the limited number of publications in other sub-systems, their collaboration strengths with the biophysical sub-system reduced dramatically, with the largest (CS = 21.6%) being between the biophysical and economic sub-systems. 80% of the total CSs between these two sub-systems were contributed by the top 10 biophysical disciplines and the economic disciplines related to technology/engineering and agriculture. Some collaboration strengths were established between the biophysical and climatic sub-systems (6.9%), and the biophysical and governance sub-systems (8.9%), whereas the collaboration strengths in other sub-systems were negligible (CS < 3%).

During the 1984–2000 period, the research collaboration in disciplines within and between sub-systems were all larger than 50%, with the most connected newly developed disciplines including Evolutionary Biology (B), Genetics and Heredity (E), Mathematical Sociology (S), and Health Policy and Services (G). The largest three research collaborations were between the biophysical and climatic sub-systems (*C* = 96%), between the climatic and governance sub-systems (*C* = 95%), and between the climatic and societal sub-systems (*C* = 94%). The strongest CS remained unchanged within the biophysical sub-system, which also showed the greatest increase of 18.5% from the previous period. The research focuses have been shifted from interactions between Environmental Sciences and Water Resources to interactions between Environmental Sciences and Marine and Freshwater Biology. Along with Physical Geography, Biodiversity Conservation, and Zoology, these disciplines contributed to about 75% of total CSs within the biophysical sub-systems. There were also slight increases in CS between the biophysical and economic sub-systems (2.6%), whereas the highest reduction in CS were between the biophysical and governance sub-systems (− 8.2%), followed by between the biophysical and climatic sub-systems (− 4.3%).

During the 2001–2017 period, average C reached about 90% among all sub-systems as the discipline system continued to mature. The CS continued to concentrate within the biophysical sub-system with the greatest increase of 12.9%. There were also slight increases between the biophysical and climatic (2.3%), economic and climatic (0.4%), and between the biophysical and societal (0.3%) sub-systems, corresponding to increase research collaborations among Environmental Sciences, Meteorology, Environmental Engineering, and Geography (Human) disciplines. However, CS between the biophysical and economic sub-systems experienced the largest reduction (− 2.5%) during this period, followed by those within the economic (− 1.2%), between the biophysical and governance (− 0.2%), and between the economic and governance (− 0.1%) sub-systems. On the other hand, there was also no change in cross-disciplinary CS among the societal, governance, and climatic sub-systems.

### Temporal change of research collaborations on management issues

For research collaborations on management issues (Fig. 4, and for Supplementary Information B for most connected issues in the sub-systems), the average C among all sub-systems were about 47% during the 1900–1983 period, particularly for the biophysical sub-system with the economic (75%), climatic (65%), and governance (64%) sub-systems and within the economic sub-system (68%). These interactions were mainly contributed by the major topics: “pollution and treatment”, “energy”, “management and control”, “other climatic extreme”, and “agriculture and irrigation”. Collaborations within the biophysical sub-system remained the strongest (*C* = 79%, CS = 79.3%), with the top 7 management issues (“pollution and treatment”, “water scarcity and availability”, “salinity, acidification and alkalinity”, “ecological degradation and restoration”, “hazards and their mitigation”, “erosion and sedimentation”, and “flood and drought and their mitigation”) contributing to over 75% of the total CS within the biophysical sub-system. Research strengths were also highly located between the biophysical and economic sub-systems (CS = 46.2%), 75% of which were contributed by about half of the economic issues (most dominantly “energy”, “agriculture and irrigation”, “transportation” issues); and between the biophysical and governance sub-systems (CS = 31.1%), 75% of which were contributed by the top 50% of governance issues (most dominantly “management and control”, “government”, and “forecasting” issues). Then, the CSs diminished quickly among sub-systems because of limited number of publications.

During the 1984–2000 period, with increasing recognitions on the inter-system issues, interactions among all the sub-systems have almost been established (average *C* = 95%). CS within the biophysical sub-system continued to intensify with the greatest increment of 13.3%, with the most interacted topics being changed from between “water use and supply” and “pollution and treatment” to between “ecological degradation and restoration” and “pollution and treatment”. Like the previous period, top 8 out of the total 18 issues contributed to over 75% of the total CS within the biophysical sub-system, with “pesticide and fertilization” as the newly emerged major issue. Major increases in CS were also identified between the biophysical and societal sub-systems (6.4%), 75% of which were contributed by the top 40% societal issues, with major issues “social event”, “transition” and “gender” replacing “civilisation” and “technology development”. The third increase in CS was between the biophysical and climatic sub-system (4.5%), most dominantly by “climate change”, “other climatic extremes”, and “temperature rise” issues. Moreover, CSs between and within the economic sub-systems (− 10.6% within E and − 8.9% between E–B) and between and within the governance sub-system (− 1.9% within G and − 2.3% between G–E) experienced reductions.

During the 2001–2017 period, with collaborations on management issues among all sub-systems being further established (average *C* = 96%). The gaining collaboration strengths were most realised between the biophysical and climatic sub-systems (with “land use and land cover change” as the newly emerged major biophysical issues), and between the biophysical and governance (with “mapping and tool” and “operation” as newly emerged major governance issues) sub-systems (increased by 6.6% and 4.5%, respectively). CS between the biophysical and economic sub-systems mostly dominated by “ecological degradation and restoration” and “agriculture and irrigation” continued to demonstrate high reduction (-2.5%), while changes in other cross-system issues remained relatively low and the most connected issues remained unchanged (about 1% changes in CSs). There were also changes of the most connected issues in the economic sub-system (with “industry” and “population migration” replacing “mining” and “textile and paper mill” issues) and the societal sub-system (“knowledge and capacity” and “technology development” as the newly emerged major issues) (refer to Supplementary Information C for more details).

### Spatial distribution of collaboration in terms of disciplines and management issues

**Fig. 5:**
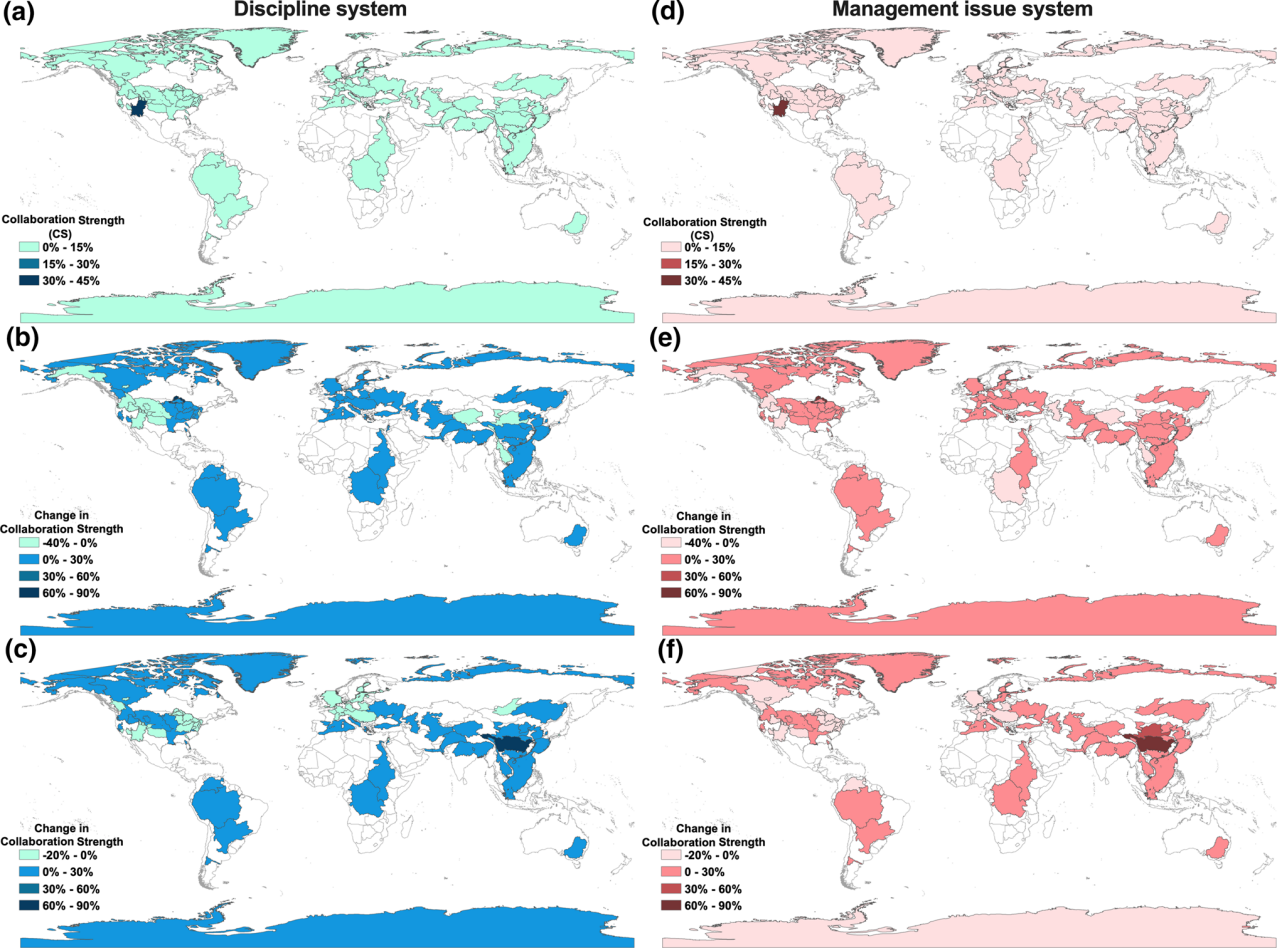
The initial Collaboration Strengths (CS) for the discipline systems of 95 river basins during **a** 1900–1983, and changes of their CSs during **b** 1984–2000 and **c** 2001–2017; the initial Collaboration Strengths (CS) for the management issue systems of 95 river basins during **d** 1900–1983, and changes of their CSs during **e** 1984–2000 and **f** 2001–2017

In terms of collaborations on disciplines (Fig. 5a, b, c), most river basins (83%) have low to no collaboration strength (CS < 5%) during the 1900–1987 period. This spanned major river basins in Asia (e.g., the Mekong River), Europe (e.g., the Mediterranean Sea), America (e.g., the Mississippi River) and Africa (e.g., the Nile River). Even though for the Colorado River, Lake Ontario, and the Mississippi River, the top 3 river basins identified to have the highest research collaborations during this period, their average CS was only 20%. During the 1984–2000 period, almost all river basins (78%) experienced increase in collaboration strength. The top 3 increases in CS were the Great Lakes, the Chesapeake Bay, and the Mississippi River, with an average increase in CS of over 30%. This was reversed for most of the high CS river basins in the previous period, with the largest reduction experienced by the Colorado River (− 40%), the Green River (− 3%), and the Yukon River (− 1%). In the final 2001–2017 period, further growths were identified for 60% of all river basins, with reductions mostly in the North American and European river basins: The River Rhine, the Chesapeake Bay, and the Lake Ontario being the top 3 and with an average reduction in CS of − 12%. Conversely, Asian rivers demonstrated strong growth in CS, especially the Yangtze River (70%), the Yellow River (30%), and the Pearl River (20%). The collaborations were mainly focused on those with the biophysical sub-system for all river basins. The average disciplinary connections with the biophysical sub-system increased from about 18% initially to 45% and reaching 47% in the final period. Only 4% on average were contributed by the connections with the societal sub-systems, with the Death Valley in Antarctica being the most focused (17%).

For collaborations on management issues (Fig. 5d, e, f), even more river basins (95%) demonstrated low to no collaboration strength (CS < 5%) during the 1900–1984 period, which spanned major river basins across Asia, America, Europe, Africa, Arctics, and Oceania (e.g., the Yangtze River, the Great Lakes, the Danube River, the Nile River, the Arctic Lakes, and the Murray-Darling River). The top 3 river basins were in North America and Europe (the Colorado River, the River Rhine, and the Lake Ontario), which only had an average CS of 20%. In the following period, over 85% of rivers experienced increases in CS. As the Great Lakes demonstrated the highest increase in CS (85%), the following Mississippi River and the River Rhine only increased by 18% and 13%, respectively. Most of the river basins that experienced decreases in CS were in Asia, Africa, and North America, especially the Colorado River (− 37%), but diminished quickly among the following Mekong River (− 1%), and the River Meuse (− 0.4%). The trend of reduction has been propagated to more rivers (over 40%) during 2001–2017, with the top 3 being the River Rhine, the Lake Ontario, and Lake Erie. Like the discipline system, increases in CS were most evident in the Asian river basins, especially the Yangtze River (91%), the Yellow River (37%) and the Pearl River (23%). The issue connections within the biophysical sub-system continued to dominate in all river basins, with slightly lower average collaboration intensities (CS increased from 16% during the initial period and remained 39% during the last two periods) than that in the disciplinary connections, whereas greater collaboration intensities were also identified on average for the societal (10%, highest by the Death Valley) and governance (21%, highest by the Murray-Darling River) sub-systems.

## Discussion

This study used 95 most researched river basins as an example to investigate the research collaborations in the Earth system since 1900. We defined the river basin as a complex system comprising of five interactive sub-systems: the biophysical (B), economic (E), societal (S), climatic (C) and governance (G) sub-systems. Based on the disciplines involved and the management issues studied, the extent of research collaborations were measured by C (establishment of collaboration by existence of publication) and CS (the strength of collaboration by number of publications) among the five sub-systems. Key results are summarised below:

The development of knowledge on global river basins in terms of publications experienced preliminary development (1900–1983), rapid development (1984–2000), and stabilisation (2001–2017). The biophysical and economic sub-systems for the discipline system (75% and 18% of total publications, respectively) and the management issue system (53% and 17% respectively) dominated the whole study period. The top ten disciplines in the biophysical sub-system contributed a majority (over 60%) of publications, dominated by Environmental Sciences, Water Resources/Hydrology, and Ecology during the study period. Interdisciplinary collaboration strength (CS) was also concentrated within the biophysical sub-system, whereas disciplinary CSs related to the societal and governance sub-systems were lower than 1%. On the other hand, more diverse focuses were observed in the management issue system. CSs between the governance and biophysical sub-systems in management issues system (32.8% on average) was much larger than the discipline system (3.4% on average) in all periods. With lower CSs in the biophysical-economic sub-systems for management issues, there were also increases in management issues and decrease in disciplines for collaborations related to the climatic, governance and societal sub-systems in 2001–2017 (Fig. 4). From a spatial perspective, it was a global tendency for river basins to have the most focus on the biophysical and economic sub-systems, implying a unified, non-diverse knowledge development pattern among river basins (Fig. 3). However, the distribution of collaborations strength (CS) was extremely uneven, mainly driven by large river basins (e.g., the Yangtze River and the Great Lakes) and dominated by the biophysical sub-system (Fig. 5).

There are both intrinsic and extrinsic causes to these findings. Firstly, knowledge development is a path-dependent process (Coccia 2020). Research on river basins is rooted in understanding of the biophysical environment, and it is natural that initial research collaborations occurred among related biophysical disciplines and further propagated to the economic disciplines due to their quantitative approaches. However, it is more difficult to develop research collaborations between natural and (non-economic) social sciences, as there are intrinsic hinderances between the two both ontologically and epistemologically (Ludwig and El-Hani 2020). Social sciences are based on umpteen variety of materials, in-depth analyses, and qualitative models, thus, there exists a deep gulf with natural science characterized by quantitative approach and formalized model (Cash et al. 2003; Kinnebrew et al. 2020). Practically, when there is the ethnical or political push for integrating social sciences in natural resources management, it is a common practice to include one or two economists and rarely other social scientists as committee members or advisors (Bennett et al. 2017; Charnley et al. 2017; Ludwig and El-Hani 2020); or social perspectives are usually considered directly through public debate and public engagement rather than involvement of social sciences (Ounanian et al. 2013). Secondly, extrinsic dynamics does not encourage a whole-of-system understanding among different disciplines, further hindering the development of research collaborations. Academic capitalism (market and market-like activities) has resulted in that universities and their members engage into and generate external revenue from research and service activities (Slaughter and Rhoades 2004). There has also been a push by university policy makers to leverage academic assets toward regional and national economic growth (Callon 1994; Rasmussen et al. 2017). Research from social sciences are neither of these two cases. In addition, the metrics-driven evaluation of scientific activities in which natural science journals generally have much higher impact factors than those in social sciences, which could further marginalise social science studies (Rasmussen et al. 2017; Muller 2018).

Our findings help explain global river basins degradations from the perspective of knowledge development. It was indicated that research collaborations on river basins have been dominated and continued to intensify for disciplines and management issues related to the biophysical sub-system, its links with the economic, and more recently with the climatic sub-system. However, both the disciplines and management issues related to the societal sub-system have been consistently neglected since 1900. This has led to limited capacity in explaining the primary driver of human behaviours, thus imbalanced human-nature relationship in the Anthropocene (Wei et al. 2018). Similarly for the governance sub-system, there was very limited research collaboration in the discipline system compared to that in the management system which implies that government issues were addressed by biophysical disciplines rather than the core governance disciplines within social sciences (e.g. Law, International Relations, Political Science). While the governance sub-system must coordinate the interactive economic and biophysical sub-systems on one hand, it is constructed and rooted in the societal sub-system on the other hand. With the limited involvement of governance in social science, we have never been at a position to properly address the unsustainable challenges. Furthermore, the unified, non-diverse knowledge pattern among all river basins could not support understanding through specialised, complementary knowledge development (Foray 2018). Finally, as knowledge development entered the stabilization stage (McDonald et al. 2018; Bhattacharya and Packalen 2020; Park et al. 2021), if current research collaboration patterns continued, we would have much weaker capacity to reverse the trend that global river basins will face further deterioration in future.

Our findings can assist strategic planning and management of cross-disciplinary research collaboration on the Earth system for improving the governance capacity in modifying the relationship between human and nature under climate change in the Anthropocene. By considering river basins as complex systems and as crucial links to all other cycles of Earth systems, we uniquely defined a river basin system as five sub-systems: the biophysical, economic, societal, governance, and climatic sub-systems and analysed the research collaboration between them using network approach. Based on the blind spots and weakness of research collaboration within and between any of the sub-systems identified in this study, we give the following two suggestions for pattern shifts of research collaboration. First, research on the means and processes of knowledge development should be strengthened as it is the basis for strategical planning and management of research. This suggestion is given as the means and processes of scientific research have not always been central to scientific activities, and strategic planning and management of research tend to rely on scientists’ post hoc reflections which often lack the whole-of-system understanding (Cash et al. 2003; Coccia 2020). This has resulted in that planning of many disciplines is still practiced within what we characterize as a closed knowledge system: self-regulated, organized, and agenda setting within certain disciplinary boundaries (Kuhn 1996; Holling 2001). Second, research on the societal sub-system should be moved to the centre of Earth System science development, allowing the structural change of current interactions among different sub-systems (Ison and Wei 2017; Bhattacharya and Packalen 2020). Nobel Laureate Herbert Simon held that social sciences, and not the natural sciences, should be labelled the “hard sciences” (Huppatz 2015). This is because the dynamic nature of variables that influences human cognition and the intricate web that drives human behaviours are much more difficult to quantify than biophysical variables in the natural environment. In fact, understanding systems change dynamics in human society remains in many ways a mystery. Therefore, besides global initiatives for strengthening cross-disciplinary research collaboration and advocation of the shift of knowledge production pattern to the “Quadruple Helix”, we call for a revolution in the governance of science development to transform the current pattern of knowledge development.

The limitations of this study should be recognised. Bibliographic databases like the WoS classify journals not publications along coarse disciplinary boundaries, and there are blurring of disciplinary boundaries due to increasing cross-disciplinary research that require better delineation of the diverse spectrum of scientific knowledge in future (Glänzel and Schubert 2003; Neuhaus and Daniel 2009). In additions, only academic publications including “river basin” and its equivalents, published in English were studied.

## Conclusion

To conclude, the ability of the human society to cope with global challenges in the Anthropocene will critically depend on the systemic development of its knowledge. Science profits from a continuous process of self-reflection. Our findings are expected to assist more precise, efficient, and predicable strategic management on research collaboration for a sustainable Earth System.

## Supplementary Information

Below is the link to the electronic supplementary material.

Supplementary file1 (PDF 171 kb)

## Data Availability

Data and codes pertaining to this work is available from https://doi.org/10.5281/zenodo.5219305.

## Footnotes

1 https://images.webofknowledge.com/images/help/WOS/hp_subject_category_terms_tasca.html.

2 https://images.webofknowledge.com/images/help/WOS/hp_research_areas_easca.html.

3 https://clarivate.com/derwent/zh-hans/solutions/derwent-data-analyzer-automated-ip-intelligence/.
