# Supplementary material for: The gulf of cross-disciplinary research collaborations on global river basins is not narrowed
Source: Ambio. 2022 Mar 23;51(9):1994–2006. doi: 10.1007/s13280-022-01716-0 (PMC9287508; doi:10.1007/s13280-022-01716-0)
Supplement: Supplementary file 1 — Supplementary file1 (PDF 171 kb) [file 13280_2022_1716_MOESM1_ESM.pdf]

Ambio

Supplementary Information

This supplementary information has not been peer reviewed.

Title: **The gulf of cross-disciplinary research collaborations on global river basins is not narrowed**

A. Classifications of sub-systems for disciplines and management issues

Table A.1 and A.2 summarised the disciplines and management issues classified under each of the five sub-systems, respectively.

Table A.1 The disciplines classified in each sub-system

**Sub-systems**	**ISI classified disciplines**
Biophysical sub-system	Environmental Sciences<br>Water Resources<br>Marine & Freshwater Biology<br>Geosciences, Multidisciplinary<br>Ecology<br>Limnology<br>Oceanography<br>Geography, Physical<br>Biodiversity Conservation<br>Environmental Studies<br>Zoology<br>Soil Science<br>Geochemistry & Geophysics<br>Plant Sciences<br>Evolutionary Biology<br>Biochemistry & Molecular Biology<br>Chemistry, Analytical<br>Geology<br>Microbiology<br>Biology<br>Paleontology<br>Biotechnology & Applied Microbiology<br>Chemistry, Multidisciplinary<br>Ornithology<br>Entomology<br>Veterinary Sciences<br>Statistics & Probability<br>Nuclear Science & Technology<br>Parasitology<br>Mathematics, Interdisciplinary Applications<br>Biochemical Research Methods<br>Chemistry, Inorganic & Nuclear<br>Mechanics<br>Chemistry, Applied<br>Chemistry, Physical<br>Thermodynamics

	Instruments & Instrumentation<br>Mineralogy<br>Physics, Multidisciplinary<br>Spectroscopy<br>Electrochemistry<br>Mathematics, Applied<br>Physics, Mathematical<br>Cell Biology<br>Biophysics<br>Physics, Fluids & Plasmas<br>Physics, Applied<br>Astronomy & Astrophysics<br>Developmental Biology<br>Physics, Nuclear<br>Nanoscience & Nanotechnology<br>Chemistry, Organic<br>Physics, Atomic, Molecular & Chemical<br>Mathematical & Computational Biology<br>Mycology<br>Optics<br>Reproductive Biology<br>Chemistry, Medicinal<br>Acoustics<br>Physics, Condensed Matter<br>Physics, Particles & Fields<br>Microscopy<br>Crystallography<br>Mathematics
Economic sub-system	Engineering, Environmental<br>Engineering, Civil<br>Fisheries<br>Toxicology<br>Agronomy<br>Public, Environmental & Occupational Health<br>Green & Sustainable Science & Technology<br>Remote Sensing<br>Genetics & Heredity<br>Forestry<br>Agriculture, Multidisciplinary<br>Engineering, Chemical<br>Energy & Fuels<br>Agricultural Engineering<br>Imaging Science & Photographic Technology<br>Urban Studies<br>Computer Science, Interdisciplinary Applications<br>Engineering, Geological<br>Food Science & Technology<br>Engineering, Mechanical<br>Engineering, Ocean<br>Materials Science, Multidisciplinary<br>Engineering, Multidisciplinary<br>Horticulture<br>Transportation<br>Engineering, Electrical & Electronic<br>Infectious Diseases<br>Tropical Medicine<br>Agriculture, Dairy & Animal Science<br>Construction & Building Technology<br>Transportation Science & Technology<br>Radiology, Nuclear Medicine & Medical Imaging

	Physiology<br>Computer Science, Information Systems<br>Engineering, Industrial<br>Endocrinology & Metabolism<br>Immunology<br>Hospitality, Leisure, Sport & Tourism<br>Mining & Mineral Processing<br>Computer Science, Artificial Intelligence<br>Pharmacology & Pharmacy<br>Engineering, Marine<br>Metallurgy & Metallurgical Engineering<br>Engineering, Petroleum<br>Medicine, General & Internal<br>Nutrition & Dietetics<br>Health Care Sciences & Services<br>Architecture<br>Materials Science, Paper & Wood<br>Medicine, Research & Experimental<br>Computer Science, Software Engineering<br>Engineering, Manufacturing<br>Medicine, Legal<br>Virology<br>Polymer Science<br>Materials Science, Characterization & Testing<br>Computer Science, Theory & Methods<br>Automation & Control Systems<br>Neurosciences<br>Pathology<br>Psychology, Multidisciplinary<br>Pediatrics<br>Psychiatry<br>Sport Sciences<br>Dentistry, Oral Surgery & Medicine<br>Telecommunications<br>Oncology<br>Audiology & Speech-Language Pathology<br>Materials Science, Ceramics<br>Psychology, Clinical<br>Nursing<br>Psychology, Educational<br>Substance Abuse<br>Surgery<br>Respiratory System<br>Otorhinolaryngology<br>Anesthesiology<br>Ergonomics<br>Allergy<br>Computer Science, Hardware & Architecture<br>Urology & Nephrology<br>Ophthalmology<br>Anatomy & Morphology<br>Clinical Neurology<br>Primary Health Care<br>Psychology, Social<br>Psychology<br>Emergency Medicine<br>Psychology, Developmental<br>Obstetrics & Gynecology<br>Integrative & Complementary Medicine<br>Materials Science, Textiles

	Geriatrics & Gerontology<br>Materials Science, Biomaterials<br>Engineering, Aerospace<br>Medical Ethics<br>Critical Care Medicine<br>Dermatology<br>Psychology, Applied<br>Psychology, Experimental<br>Engineering, Biomedical<br>Gastroenterology & Hepatology<br>Peripheral Vascular Disease<br>Computer Science, Cybernetics<br>Gerontology
Societal sub-system	Geography<br>Anthropology<br>Planning & Development<br>Archaeology<br>Sociology<br>Area Studies<br>Social Sciences, Interdisciplinary<br>History<br>History & Philosophy Of Science<br>Social Sciences, Mathematical Methods<br>Information Science & Library Science<br>History Of Social Sciences<br>Behavioral Sciences<br>Education & Educational Research<br>Demography<br>Social Sciences, Biomedical<br>Communication<br>Cultural Studies<br>Women's Studies<br>Ethics<br>Linguistics<br>Ethnic Studies<br>Education, Scientific Disciplines<br>Social Issues<br>Language & Linguistics<br>Art<br>Criminology & Penology<br>Family Studies<br>Asian Studies<br>Humanities, Multidisciplinary<br>Film, Radio, Television<br>Social Work<br>Religion<br>Logic
Climatic sub-system	Meteorology & Atmospheric Sciences<br>Multidisciplinary Sciences
Governance sub-system	Economics<br>Political Science<br>Law<br>International Relations<br>Operations Research & Management Science<br>Agricultural Economics & Policy<br>Management<br>Public Administration<br>Business<br>Health Policy & Services<br>Industrial Relations & Labor

	Business, Finance

Table A.2 the sub-topics for all key words and their corresponding sub-systems.

**SES sub-system**	**Management issue topics**
Biophysical sub-system	Ecological degradation and restoration<br>Pollution and treatment<br>Flood, drought and their mitigation<br>Erosion and sedimentation<br>Water use and supply<br>Salinity, acidification and alkalinity<br>Other hazards and their mitigation<br>Land use and land cover change<br>Pesticide and fertilisation<br>Hydrological change<br>Sea surface change<br>Biodiversity<br>Carbon emission and sequestration<br>Afforestation and deforestation<br>Drinking water and salinisation<br>Natural resources other than land and water<br>Geological change<br>Climate change mitigation and adaptation
Economic sub-system	Agriculture and irrigation<br>Urban issue<br>Hydropower<br>General economic development<br>Human activity<br>Transportation<br>Construction<br>Aquaculture and fishery<br>Energy<br>Population migration<br>Public health<br>Industry<br>Tourism and recreation<br>Food security<br>Mining<br>Rural issue<br>Pharmacy<br>Human health<br>Emergency<br>Forestry<br>Textile and paper mill<br>Religion
Societal sub-system	Population<br>Value<br>History<br>Conflict<br>General societal issue<br>Behaviour<br>Transition<br>Technology development<br>Knowledge and capacity<br>Social event<br>Equality<br>Globalisation<br>Gender<br>Education and training<br>Class and ethnicity

	Media and communication<br>Public affairs other than health<br>Relation<br>Civilisation<br>Employment<br>Citizenship<br>Literature and language<br>Power<br>Arts and aesthetics<br>Crime
Climatic sub-system	Climate change<br>Climatic extremes other than floods and droughts<br>Precipitation change<br>Temperature rise<br>Greenhouse gas increase<br>Socio-ecological<br>Science-policy<br>Sustainability
Governance sub-system	Management and control<br>Risk and impact assessment<br>Plan and strategy<br>Regulation/rule<br>Policy<br>Organisations and agencies<br>Decision making<br>Monitoring<br>Forecasting<br>Trading and entitlement<br>Mapping and tool<br>Governance<br>Law<br>Operation<br>Cooperation<br>Standard/guideline/criteria<br>Politics<br>Stakeholdering<br>Tax and subsidy<br>Prospect and vision<br>Permit/certificate

B. Most connected disciplines and management issues for each sub-systems

Table B.1, B.2, and B.3 summarised the most connected disciplines and management issues during the three periods: 1900-1983, 1984-2000, and 2001-2017, respectively.

Table B.1 The most connected discipline (top) and management issues (bottom) during 1900 – 1983.

B	Env. Science-Water Resource<br>Water scarcity-Pollution				
E	Env. Engineering-Env. Sci.<br>Energy-Pollution	Envi. Engineering-Civil Engineering<br>Agriculture-Energy			
G	Law-Water Resources<br>Manag. and control-Pollution	Law-Civil Engineering<br>Energy-Manag. and control	International Relations-Law<br>Trading and entitlement-Manag. and control		
S	Inter. Social Sciences-Env. Sci.<br>Behaviour-Pollution	Geography-Multi. Agriculture<br>History-Agriculture; Population-Agriculture	Geography-Economics<br>Behaviour-Management and control	History-Geography<br>Population-History	
C	Multi. Sci.-Envi. Sciences<br>Other climatic extreme-Pollution	Multi. Sci.-Civil Eng.<br>Other climatic extreme-Energy	Multi. Sci.-Law<br>Other climatic extreme-Manag. and control	Multi. Sci.-Inter. Social Science<br>Other climatic extreme-History	Multi. Sci.-Meteorology<br>/
	B	E	G	S	C

Table B.2 The most connected discipline (top) and management issues (bottom) during 1984 – 2000.

B	Marine & Freshwater Biology-Env. Sci.

	B	E	G	S	C
	Ecological degradation-Pollution				
E	Env. Engineering-Env. Science<br>Agriculture-Pollution	Env. Engineering-Toxicology<br>Transportation-Agriculture			
G	Economics-Environmental Science<br>Pollution-Manag. and control	Economics-Env. Engineering<br>Agriculture-Manag. and control	Agricultural Economics &Policy-Economics<br>Risk assessment – Manag. and control		
S	Geography-Env. Sci.<br>Population-Ecological degradation	Geography-Env. Engineering<br>Population-Agriculture	Geography-Economics<br>Population-Management and control	Planning & Development-Geography<br>Population-History	
C	Meteorology-Env. Science<br>Climate change-Pollution	Meteorology-Env. Eng.<br>Climate change-Agriculture	Meteorology-Economics<br>Climate change-Management and control	Meteorology-Geography<br>Climate change-Population	Multi. Sci.-Meteorology<br>Other climatic extreme-Climate change

Table B.3 The most connected discipline (top) and management issues (bottom) during 2001 – 2017.

	B	E
B	Marine & Freshwater Biology-Env. Sci.<br>Ecological degradation-Pollution	
E	Env. Engineering-Env. Sci.<br>Agriculture-Ecological degradation	Env. Engineering-Toxicology<br>Hydropower-Agriculture

G	Economics-Env. Sci.	Economics-Env. Engineering	International Relations-Economics		
	Ecological degradation-Manag. and control	Agriculture-Manag. and control	Risk assessment – Manag. and control		
S	Geography-Env. Sci.	Geography-Env. Engineering	Geography-Env. Sci.	Planning & Development-Geography	
	Population-Ecological degradation	Population-Agriculture	Population-Ecological degradation	Conflict-Population	
C	Meteorology-Env. Sci.	Meteorology-Env. Engineering	Meteorology-Economics	Meteorology-Geography	Multi. Sci. - Meteorology
	Climate change-Ecological degradation	Climate change-Agriculture	Climate change-Management and control	Climate change-Population	Other climatic extreme-Climate change
	B	E	G	S	C

C. Additional results

Comparison of research collaboration between disciplines and management issues

During the initial period (1900-1983), the research collaborations (C) of management issues within B, within E, B-E, B-S, and E-S sub-systems were much larger than that for the disciplines. Similarly, the collaboration strengths (CS) of management issues within B, within E, within G, between B-E, between B-S, between E-S, between B-G, between G-E sub-systems were much larger than that for the disciplines. For example, CS of management issues between the B-G sub-systems on management issues (31.1%) was much larger than that of the disciplines (8.9%). It was identified that the central management focus was between “water use and supply” and “pollution and treatment”, which was mainly addressed by the Environmental Sciences and Water Resources disciplines.

During the 1984-2000 period, there were consistent increases of research collaboration (C) for both the discipline system and the management issue system. However, there appeared contrary trends in CS between the discipline system and the management issue system in B-E (increase in disciplines and decrease in management issues). More commonly, there were increase in management issues and decrease in disciplines, especially those related to the societal, climatic and governance sub-systems. It was identified that the central management focus was changed to “ecological degradation and restoration” and “pollution and treatment” issues, the most connected disciplines during the 1984-2000 period were between the Marine & Freshwater Biology and Environmental Sciences.

During the 2001-2017 period, the research collaboration (C) within and between all sub-systems were already very high. Decreases in management issues but increases in disciplines were most commonly observed, especially those related to the economic sub-system (e.g., within E, B-E, C-E, G-E). Regarding the CS, there were decreases in management issues and increases/unchanged in disciplines for those in the societal sub-system, but increases in management issues and decreases or unchanged in disciplines for those in the governance and climatic sub-system (except G-S, C-S). In particular CS between the governance sub-system and the biophysical subsystem on management issues continued to increase (Figure C.1).

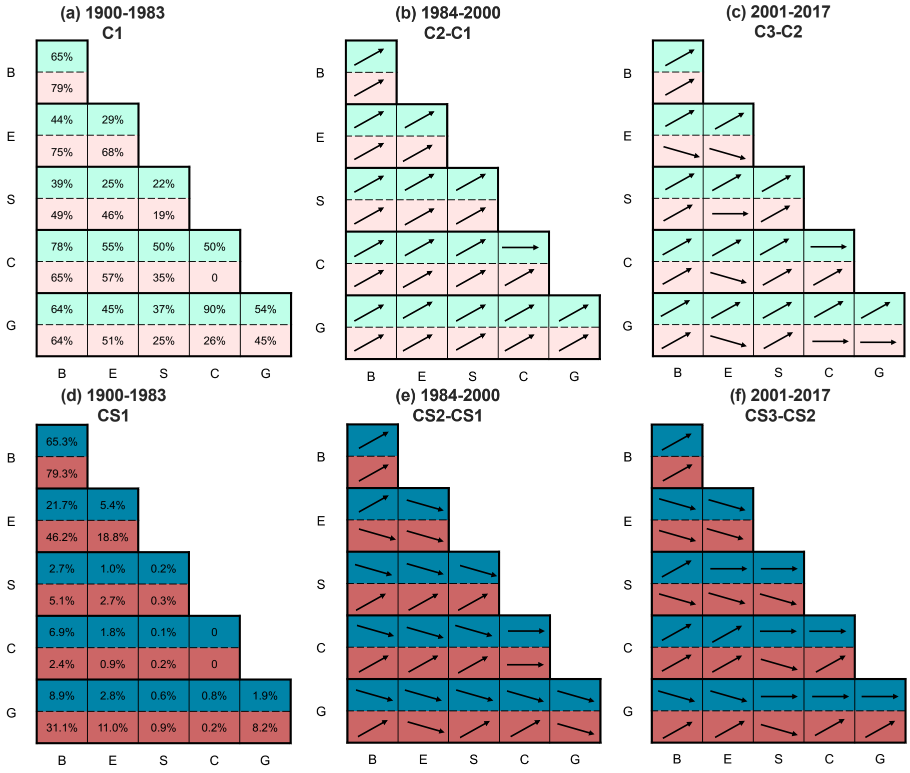

Figure C.1 Comparisons between the discipline system (blue) and management issue system (orange) for **(a)** collaboration (C) during 1900-1983; **(b)** change trend of C during 1984-2000; and **(c)** change trend of C during 2001-2017; comparisons for **(d)** collaboration strengths (CS) during 1900-1983; **(e)** change trend of CS during 1984-2000; and **(f)** change trend of CS during 2001-2017.
